# “Being able to do whatever you wanna do as a woman is important:” a qualitative exploration of contraception as a frame of reference for HIV prevention with PrEP

**DOI:** 10.1186/s12978-022-01393-y

**Published:** 2022-04-07

**Authors:** Emma Chew Murphy, Antoinette Danvers, Andrés Ramírez Zamudio, Karina Avila, Meghan Proehl, Tatiana Gonzalez-Argoti, Joanne E. Mantell, Laurie J. Bauman, Siobhan M. Dolan

**Affiliations:** 1 Department of Obstetrics & Gynecology and Women’s Health, Montefiore Medical Center/Albert Einstein College of Medicine, Bronx, NY USA; 2 Department of Obstetrics, Gynecology and Reproductive Science, Icahn School of Medicine at Mount Sinai and Mount Sinai Health System, New York, NY USA; 3 HIV Center for Clinical and Behavioral Studies, Gender, Sexuality and Health Area, Department of Psychiatry, Columbia University Irving Medical Center, New York, NY USA; 4 Departments of Pediatrics and Psychiatry and Behavioral Sciences, Albert Einstein College of Medicine, Bronx, NY USA; 5 Icahn School of Medicine at Mount Sinai and Mount Sinai Health System, 1176 Fifth Avenue, 9th Floor, Box 1170, New York, NY 10029 USA

**Keywords:** PrEP, HIV prevention, Birth control, Sexual health, Sex positivity

## Abstract

**Background:**

Use of HIV PrEP (pre-exposure prophylaxis) is a strategic tool in the effort to end the HIV epidemic. 20% of new HIV infections in the US are among cis-gender women, yet they comprise only 5% of all PrEP users. Black women disproportionately bear the burden of new HIV acquisition and accounted for almost 60% of new HIV diagnoses among women in 2018. Increasing understanding and uptake of PrEP among women at risk of HIV acquisition in alignment with their reproductive values and preferences is key to increasing PrEP uptake and decreasing HIV burden in this population.

**Objective:**

This study examines how experiences with contraception among women of color shape their perceptions and preferences regarding HIV PrEP to inform counseling that aligns with their reproductive values.

**Methods:**

Women aged 18–45 who self-identified as Black or Latina were recruited at an academic medical center in the Bronx from June 2018 to July 2019. We enrolled 30 participants seeking family planning care (10), prenatal care (10), or care for sexually transmitted infections (10). Participants completed a brief written survey assessing their risk of HIV acquisition. Semi-structured, face-to-face interviews were then audio-recorded, transcribed, and entered into Dedoose. Grounded theory and constant comparison approaches were used to analyze the data.

**Results:**

Twenty-one participants (70%) screened positive for HIV acquisition risk. Four had received information on PrEP from a medical provider prior to the interview. Three themes emerged from the qualitative analysis: (1) Similar to oral contraception, women conceptualized PrEP as a “daily pill” to support their reproductive health; (2) Women perceived PrEP as a tool to support autonomy and pleasure in their sexual health; (3) Like birth control, women desired multiple delivery options for HIV prophylaxis.

**Conclusions:**

Contraception may serve as a frame of reference when counseling about PrEP among cis-women at risk of acquiring HIV. Our study suggests that this approach re-contextualizes counseling on PrEP within a sex-positive framework that prioritizes pleasure, safety, and autonomy as integral to sexual and reproductive wellness. Consideration of historically marginalized women’s experiences with contraception and reproductive values may facilitate their use of PrEP.

## Plain Language Summary

PrEP (pre-exposure prophylaxis) is a medicine taken daily by people at risk of getting HIV from sex or injection drug use. Although PrEP is a safe and effective medication for women, the use of PrEP remains exceedingly low among cis-gender women at risk of HIV in the US. This study examines how experiences with contraception among women of color, who disproportionately bear the burden of HIV acquisition, shape their perceptions and preferences regarding PrEP. We interviewed 30 women who self-identified as Black or Latina at an academic medical center in the Bronx. Similar to oral contraception, women in this study conceptualized PrEP as a “daily pill” to support their reproductive health. This report details how women’s experiences with contraception may serve as the foundation to re-contextualize conversations on PrEP within a sex-positive framework that prioritizes pleasure, safety, and autonomy as integral to sexual and reproductive wellness.

## Introduction

HIV pre-exposure prophylaxis (PrEP) is an effective biomedical intervention that decreases the risk of HIV acquisition and is championed as a key strategy to ending the HIV epidemic. The safety and efficacy of oral emtricitabine and tenofovir disoproxil fumarate as HIV pre-exposure prophylaxis have been well-established, but women comprise only 5% of all PrEP users in the US and the adoption of PrEP remains exceedingly low among cis-gender women at risk of HIV acquisition [1]. In the US, cis-gender women are estimated to account for 25% of all people living with HIV and 20% of all new HIV infections. In a society defined by racial segregation in housing, education, income, and healthcare, Black women disproportionately bear the burden of new HIV acquisition and accounted for almost 60% of new HIV diagnoses among women in 2018 [2]. Black women are also less likely than White women to have initiated PrEP. In a 2016 analysis, 48.3% of all female PrEP users in the US were White, while 25.9% were Black and 17.5% were Latinx [3].

Increasing understanding and uptake of PrEP among women at risk of HIV acquisition are foundational to a multi-pronged strategy to decreasing the burden of HIV, particularly among women of color. Previous studies exploring women’s perceptions of PrEP have suggested that while few US women are aware of PrEP, those who are view PrEP as an important option for HIV prevention for themselves and their communities [4].

This study is part of a larger project designed to understand the receptivity of women to receiving PrEP services as part of their OBGYN care. Most women did not know about PrEP prior to the study; when we provided standardized information about it, women often contextualized PrEP through their experiences with birth control. In this analysis, we aimed to understand how women’s experiences with contraception anchor and inform their perceptions and preferences regarding PrEP.

## Methods

Participants in this study were recruited from an ambulatory care center in an urban academic center in the Bronx, New York, from June 2018 to July 2019. The care center provides the full spectrum of OBGYN services with over 155,000 visits annually. Women were eligible for the study if they were between 18 and 45 years old, spoke English, and self-identified as Black, Latina, or mixed race. Purposive sampling was used to recruit 30 participants in this study: 10 were seeking care for family planning, 10 were attending prenatal care, and 10 were seeking care for a sexually transmitted infection. Eligible participants were approached by a study coordinator after their scheduled visit was completed, and among those interested and eligible for study participation, written informed consent was obtained.

Each participant completed both a brief written survey and a semi-structured, face-to-face interview. The pre-interview survey included demographic questions and a 10-item PrEP screening tool about the participant’s sexual behaviors in the previous 12 months. The interviews were conducted by two study coordinators trained in qualitative interview techniques. The interview guide included questions on participants’ knowledge and attitudes regarding PrEP, discussions with their healthcare providers about HIV prevention, and their concerns regarding PrEP. Participants received a $50 gift card as compensation for their time after they completed the interview.

All interviews were audio-recorded, de-identified, transcribed, and entered into Dedoose [5]. The transcripts were analyzed using constant comparison and the grounded theory approach described by Auerbach and Silverstein [6]. In this process, six members of the research team reviewed the first interview to identify statements relevant to the research question. From these statements, a preliminary list of codes was developed. An additional ten interviews were then reviewed for relevant ideas, words, or phrases expressed by multiple participants to identify additional codes, which were entered into a code book. The codebook was finalized through an iterative process until consensus on all key themes was achieved. Members of the research team worked in pairs to code all transcripts. Coding discrepancies that could not be resolved by the paired coders were resolved in the larger group by consensus, which helped to ensure methodologic rigor in the coding process.

One of the salient themes that emerged from this analysis was the association between contraception and HIV prevention. For the results presented in this paper, six team members extracted, analyzed, and discussed text coded with themes and subthemes related to contraception and PrEP (Appendix). At regular intervals throughout the data analysis process, the themes were presented and reviewed by the larger interdisciplinary research team to ensure credibility and coherence in the results.

## Results

Demographic characteristics of the 30 women who participated (Table 1) reflect the population of the Bronx; 18 participants self-identified as Latina, 10 as Black/African American, and two as other. The mean age was 29.73 years (± 5.36). Seventy percent of participants screened positive for HIV acquisition risk based on the PrEP screening tool. Prior awareness of PrEP was low: four participants had learned about PrEP before their interview and none of the participants had ever been prescribed PrEP.

**Table 1:** Characteristics of the participants interviewed in the study

Characteristics	N (%)	Mean (S.D.)[Range]
Age	30	29.73 (5.36)[20–41]
Race/ethnicity		
Latinx	18 (60%)	
Black/African American	10 (33%)	
Other	2 (7%)	
Sexual orientation		
Heterosexual	25 (83%)	
Bisexual	5 (27%)	
Relationship status		
Married	8 (27%)	
Single, never married	20 (67%)	
Domestic partnership	2 (7%)	
Education		
< High school	4 (13%)	
High school/GED/Vocational training	6 (20%)	
Some college	13 (43%)	
Associate degree or higher	7 (23%)	
Number of sexual partners in the last year		1.83 (1.44)[1–6]
1–2 partners	23 (77%)	
3 or more partners	7 (23%)	
Sexually transmitted infection (STI) in the last year		
No	24 (80%)	
Yes	6 (20%)	
Number of live births among women who have ever been pregnant pregnant (n = 29)		
0 live births	8 (28%)	
> 1 live birth	21 (70%)	
HIV testing history		
Ever tested	29 (97%)	
Never tested	1 (3%)	
Positive screen for HIV acquisition risk		
Yes	21 (70%)	
No	9 (30%)	
Learned about PrEP from a medical professional		
Yes	4 (13%)	
No	25 (83%)	
Not sure	1 (33%)	
Ever been prescribed PrEPs		
Yes	0 (0%)	
No	30 (100%)	

Contraceptive use was heterogenous among the 15 women in the study who were not seeking pregnancy (Table 2).

**Table 2:** Current contraceptive use of the participants interviewed in the study

Current contraceptive use among those who do not desire pregnancy (n = 15)	N (%)
Condoms exclusively	2 (13%)
Birth control pills	4 (27%)
Depo-Provera	3 (20%)
Contraceptive implant	2 (13%)
Vaginal Ring	1 (7%)
IUD	3 (20%)
2 or more methods	4 (27%)
No method reported	1 (7%)

Women consistently referred to their experiences of contraception in their conceptualization of PrEP. Three key findings emerged from our analysis: (1) PrEP—like the contraceptive pill—was framed by women as a “daily pill” for reproductive health; (2) PrEP, as a woman-controlled option for HIV prevention, supports women’s autonomy and pleasure in their sexual health; and (3) like contraception, women desire multiple delivery options for HIV prophylaxis.

### Theme 1: “The daily pill for women”

When asked about their knowledge, perceptions, and concerns regarding PrEP, participants consistently referred to their experience with contraception as a frame of reference for the discussion. In particular, the oral contraceptive pill was a model for participants to conceptualize PrEP as “another daily pill” for their reproductive health. One participant said she would encourage her younger sister to consider taking PrEP by comparing it to her birth control pill:

We would tell my little sister…, ‘Look, you’re not settled down right now and whether you do or whether you don’t, it’s not gonna stop everything but it’ll stop [HIV] and it won’t hurt you to take it every single day just like your birth control.” (1.1)

PrEP is an easily accessible option for women because of its similarity to oral contraception—“the daily pill.” When asked how women might respond to taking a pill every day for HIV prophylaxis, one woman stated:

There’s a lot of girls who are on birth control. It’s a pill. They know what they got to do. (20.1)

Participants used their experience taking the birth control pill to assess the likelihood that they could take PrEP every day. For some, it made them more confident in their ability to adhere to a daily PrEP regimen:

So, I was on birth control pills and as many people know, the birth control pill you have to take it every day at a certain time… for me, I think it would be easy to take the pill every day, especially if it’s something I want to have as part of my lifestyle. (29.1)

In contrast, other participants noted the difficulty of adherence to a daily pill based on experience with oral contraception. As one participant stated, “I know my history from taking birth control pills, that everything going on, that I could be forgetful.” (10.1).

Others raised concern about the efficacy of PrEP based on prior experiences with the birth control pill:

‘Cause I know I used to take [birth control] and I was a forgetful person and I would have to double it up the next day.... If you miss a day out of the seven days, or miss two days out of the seven days, what’s gonna happen? (7.1)

Because nothing is really effective. On birth control they say 90 something point something, but… basically, everybody in my family [was conceived] on a pregnancy prevention pill or something, and we're just magically here anyways. (28.1)

This participant further qualified this concern by affirming that while PrEP does not confer 100% protection against HIV, PrEP was still an acceptable HIV prevention option, stating “to go from zero to 92 is better than nothing.”

### Theme 2: autonomy and pleasure in sexual health

In their comparison of PrEP and birth control, several participants highlighted both medications as within their control:

[A woman taking PrEP] has control of her health. Girl, get on that. Just like you have control of birth control pills… you do what you got to do to take care of your overall health, making a business to take that pill every day. (24.1)

They underscored the autonomy PrEP afforded to women because of the ability to take PrEP regardless of their partner’s condom use.

It’s something that would be great, because again, everything is just masculine. Wherever you go, yes, you do see condoms only for the guy. What if the guy didn’t want to [use] it, and you’re like, ‘Okay.’ You want something for yourself. You want to feel protected yourself. (16.1)

In discussing PrEP as a woman-controlled option for HIV prevention, participants framed PrEP as a tool to support women’s autonomy and pleasure in their sexual health. One participant, who equated PrEP with “girl power,” explained:

There’s a lot of things that are controlled by men. So I'm okay with [PrEP]. Tip the scale a little bit, I’m with it. (11.1)

Another participant similarly underscored women’s ability to choose to initiate PrEP as a kind of freedom over their sexual health:

[Women] can be free about it, because they can make the decision when to start [taking PrEP] and when the time is right… They just decide and go on their own. (3.1)

Several participants similarly emphasized that PrEP is a woman-controlled option and is particularly advantageous for women who desire a discreet method of HIV prevention. As one participant explained, PrEP is like most birth control in that it does not require disclosure to a sexual partner:

…you don’t have to tell them that you’re taking it… the privacy of being able to take it without anyone knowing if you don’t want them to know is amazing. (17.1)

For this participant, PrEP was directly connected to her experience of sexual pleasure. She noted that as she and her partner never use condoms due to her personal preference, PrEP would increase her sense of satisfaction and safety with sex:

[I] would find more pleasure being able to do without a condom and feel the security of, ‘Okay, I’m secure. I’m not catching any type of HIV.’

Several other participants similarly noted PrEP’s potential to promote sexual pleasure and confidence among women by alleviating their anxiety over HIV acquisition. When asked about key messaging to provide to women about PrEP, one participant suggested, “Let her know that it is okay to be who she is and we have a prevention for that without having to live in fear, be in fear of exploring that sexual part of her of who she is.” (18.1).

For another participant, PrEP was seen as a way to heighten self-confidence and self-actualization through the control and security afforded to women:

Sexual evolution. She has her—she's in full control of whether or not she catches one of the deadliest diseases out there... I think when you don't have to explain your choices, it makes you feel more secure in the choice. The stillness or the quiet part of being able to do whatever you wanna do as a woman is important… (11.1)

### Theme 3: desire for multiple options for protection from HIV

Informed by their experience with contraception, participants highlighted the desire for multiple modes to receive PrEP. Many interviewees expressed their concern about adherence to a daily pill and preferred longer-acting formulations of PrEP:

Well, me personally, I won’t remember to do it every day… It’s maybe something that we could do as a shot once a month or even once a week, or maybe like a patch, transdermal patch. (12.1)

For women, a shot would be better. You know? Because it’s easier, it’s quicker, and it’s faster, and it’s [not] something that we have to be reminded of every day when we’re busy and we’re occupied, or we’re at work. (7.1)

Several participants specifically offered their choice of a contraceptive method of longer duration as evidence of their concern about adherence to a daily pill:

For people who are on like the birth control pill, in fact they're good at this. But me, I’m like a long-term kind of person where I don’t like to think about it too often… I make an appointment for Depo every two to three months. (13.1)

That’s why I’m not on birth control [pills], this is why I was quick to do the IUD… it’s a bit much to take the pill every day. (28.1)

Other participants suggested that alternate formulations of PrEP could afford woman access to more discreet methods for HIV prevention. As one participant explained, women who desire to maintain privacy about taking PrEP might have difficulty concealing a PrEP bottle from others:

I’m thinking, if you have a bottle of pills, right, you have it. You have to physically take it, and you have to physically have it somewhere… the physical component can lead to people asking questions that you may not want to answer. (22.1)

This same participant suggested that an injectable option would be an ideal method for maintaining privacy around HIV prevention and potentially mitigating HIV-related stigma:

It’d be interesting to see if this became like a shot or something like that. People might be more willing to do it because it’s not—I feel like that also has less of a stigma... Nobody knows I’m taking something. Nobody knows it’s in my system.

Participants’ emphasis on alternate delivery modalities for PrEP underscored interest in women’s access to and control over HIV prevention.

## Discussion

This study highlights the perceptions and experiences of 30 women of color in the Bronx, a New York City borough where women bear the highest risk of HIV acquisition [7]. Despite approval in 2012, only 13% of participants had heard about of PrEP prior to the interviews and several of these women reported that they knew only of its use among men who have sex with men [8]. Still, participants drew connections to their experiences of contraception from their personal health and from women in their communities. Our results build upon prior qualitative research on the knowledge and attitudes about PrEP among women at increased risk of HIV that has affirmed women’s interest in PrEP as a relevant and important addition to their sexual healthcare [4].

Participants in our study also indicated that the conversations they hold with healthcare providers on contraception and HIV prevention—while distinct—are inextricably linked in women’s experience and understanding. Our findings suggest that women may benefit from approaches that integrate HIV prevention and family planning services. This echoes prior findings by Seidmen et al., which suggest that women are more open to counseling and initiation of PrEP in clinical settings that provide family planning care and further reinforces the need to build provider capacity for PrEP provision in such settings [9, 10].

Participants who had prior experience taking oral contraception demonstrated strong self-awareness regarding the feasibility of a daily pill for HIV prevention. While for some, prior ease with the pill bolstered their confidence in their ability to adhere successfully to PrEP, others expressed serious concern about a medication requiring daily use based on prior difficulty adhering to daily oral contraception. The standard of perfect medication adherence need not be a barrier for women interested in PrEP, but prior medication patterns are a strong predictor of an individual’s future use [11]. Provider counseling on PrEP that explores women’s experiences with taking a daily pill may support women in their decision-making and identify potential interventions to increase adherence among women who desire PrEP.

Like their birth control experiences, women want multiple delivery methods for PrEP, including longer-acting formulations. Research on choice in contraception has demonstrated that expanding options—when coupled with efforts to reduce potential barriers (including cost, lack of knowledge, and stigma)—significantly improves contraceptive uptake and continuation [12]. A variety of delivery methods for PrEP could similarly improve access, uptake, and continuation among women.

Participants’ suggestion that an injectable method may facilitate PrEP adherence and desirability among cis-gender women corroborates findings of a recent study assessing an injectable long-acting form of the HIV drug cabotegravir in sub-Saharan Africa. This study found that cabotegravir, administered as an injection once every 8 weeks, was safe and more effective than daily oral Truvada in preventing HIV transmission among cis-gender women [13]. Other PrEP agents currently in clinical development, including novel oral medications, vaginal rings, topical products, transdermal devices, implants, as well as dual modalities combining contraception and PrEP offer promise for new methods of HIV prevention [14]. Preliminary assessment of women’s preference of PrEP method among the options under review has revealed that women have diverse preferences, and their preferred agent often coincides with their current contraceptive modality [15].

In their discussion of PrEP, women identified self-determination and safety as central components of sexual pleasure. Their description of PrEP as a tool to support women’s autonomy and pleasure in their sexual health signals interest in expanding conversations on HIV prevention beyond historically fear-based approaches that have exclusively emphasized the negative consequences of sexual behavior, i.e. transmission of disease.

Participants’ interest in normalizing pleasure in their conversations on HIV prevention also indicates that women may benefit from a sex-positive framework for the counseling and provision of PrEP. In brief, sex-positivity acknowledges all consensual sexual activity as fundamentally healthy and potentially pleasurable. As a health framework, sex-positivity does not draw moral distinctions between different types of sexual activities, and shifts away from fear-based counseling toward an emphasis on increasing pleasure, health, and consent [16]. While sex-positivity has been incorporated into approaches to increasing PrEP uptake among gender and sexual minority communities, our findings suggest that a similar approach is warranted for increasing PrEP uptake among cis-women at risk of HIV acquisition [17].

Although sexual health experts and advocates have long understood the integral link between sexual health and pleasure, many health care providers are under-resourced to address the diverse experiences of sexual pleasure among their patients [18]. Our study suggests that such an approach may provide an opportunity for increased PrEP uptake among heterosexual women at risk for HIV acquisition.

There are limitations to this study. As women in our study were recruited in OBGYN clinics, their degree of access to and navigation of the healthcare system might not be generalizable to the experiences of all women at risk of HIV acquisition in the Bronx. The initial design of the semi-structured interviews did not explicitly seek to address women’s understanding of PrEP within their experience of contraception or sexual pleasure. This is an area for further study.

## Conclusion

Women’s historic experience with oral contraception informs their understanding of the limitations of a daily pill, particularly when medication efficacy depends on strict daily adherence. The obvious link between PrEP and birth control also illuminates an important cautionary lesson from the recent rise in the use of long-acting reversible contraception (LARC). Reproductive justice advocates have expressed concern that policy makers’ and healthcare providers’ enthusiasm to promote LARC use among low-income and women of color has threatened the reproductive autonomy of women at the intersection of multiple socially oppressed identities [19, 20]. Studies have shown that provider counseling on LARC is shaped by biases based on their patients’ class and race [21]. Knowledge of these biases as well as the legacy of coercion within reproductive healthcare can inform a sex-positive, patient-centered approach to increasing access to HIV prophylaxis [22].

Any promotion of PrEP among historically marginalized women should consider their experience with contraception. Whereas past and current messaging on HIV prevention messaging among cis-gender Black and Latina women has reinforced narratives that portray black and brown communities as vectors of infectious diseases, we invite healthcare policy makers and providers to recontextualize HIV prevention within a sex-positive framework that prioritizes pleasure, safety, and autonomy as integral to sexual and reproductive health and wellness.

## Data Availability

The datasets used and analyzed during the current study are available from the corresponding author upon reasonable request.

## Appendix

### Themes, sub-themes, and codes

**Table:**

Themes	Subthemes	Codes
PrEP is another “daily pill for women”	PrEP is accessible for women Adherence to birth control as a gauge for PrEP adherence	Birth control and PrEP Sexual health priorities HIV/STI prevention strategies PrEP and sex life/sexual pleasure Female controlled method Transparency/disclosure of sexual health topics PrEP and stigma PrEP and adherence PrEP and covert use PrEP willingness and receptivity
PrEP for autonomy and pleasure in women’s sexual health	Control over method Covert method Freedom in sexual expression	
Multiple delivery options for PrEP	Adherence to daily pill Longer-acting formulation Injectable PrEP	
